# Supplementary material for: The DURATIONS randomised trial design: Estimation targets, analysis methods and operating characteristics
Source: Clin Trials. 2020 Aug 16;17(6):644–53. doi: 10.1177/1740774520944377 (PMC7851720; doi:10.1177/1740774520944377)
Supplement: Quartagno_Additional_Material__-_2nd_revision-1 – Supplemental material for The DURATIONS randomised trial design: Estimation targets, analysis methods and operating characteristics [file Quartagno_Additional_Material__-_2nd_revision-1.pdf]

Table A: Results from using Model Confidence Bands method, with base-case design parameters (estimand=shortest duration non-inferior to 20 days within 10% risk difference)

In italics: scenarios for which type 1 error was not strictly controlled within 2.5%. Note the standard fractional polynomial algorithm was used here, as mfp is the only R package returning standard errors for the data.

	Acceptable Power (%)	Optimal Power (%)	Type 1 Error (%)	True Min Duration	Estimated Minimum Duration	Estimated 2.5th Perc Duration	Estimated Median Duration
Scenario 1	*89.7*	*11.7*	*10.3*	*13.1*	*10*	*12*	*16*
Scenario 2	99.2	15.2	0.8	14.5	13	15	16
Scenario 3	97.4	10	2.5	15.9	12	16	18
Scenario 4	100	79.8	0	8.0	8	8	8
Scenario 5	99.7	9.3	0.3	9.7	9	10	12
Scenario 6	97.6	8.4	2.4	10.8	9	11	13
Scenario 7	*93.3*	*23.2*	*6.7*	*16.2*	*14*	*16*	*18*
Scenario 8	*85.4*	*21.3*	*14.4*	*15.0*	*8*	*13*	*17*
Scenario 9	100	4.8	0	12.6	13	13	14
Scenario 10	100	2.5	0	15.2	16	17	18
Scenario 11	94.2	13.9	2.2	16.8	16	17	18
Scenario 12	*97*	*53.5*	*3*	*11.2*	*11*	*11*	*12*
Scenario 13	*94.8*	*46.7*	*5.2*	*8.1*	*8*	*8*	*9*
Scenario 14	*69*	*14.1*	*13.9*	*15.0*	*8*	*11*	*17*
Scenario 15	*92.2*	*11.7*	*7.8*	*12.5*	*11*	*12*	*15*
Scenario 16	99.1	14.7	0.9	12.0	11	12	14

Table B: Results from using Delta CI method, with base-case design parameters (estimand=shortest duration non-inferior to 20 days within 10% risk difference). Acceptable and optimal power, Type-1 Error, Real minimum duration, minimum, 2.5% percentile and median duration recommended. In italics: scenarios for which type 1 error was not strictly controlled within 2.5%.

	Acceptable Power (%)	Optimal Power (%)	Type 1 Error (%)	True Min Duration	Estimated Minimum Duration	Estimated 2.5th Perc Duration	Estimated Median Duration
Scenario 1	*96.6*	*13.1*	*3.4*	*13.1*	*11*	*13*	*15*
Scenario 2	*96.8*	*29.5*	*3.2*	*14.5*	*14*	*14*	*16*
Scenario 3	*95.1*	*18.9*	*3.5*	*15.9*	*13*	*15*	*17*
Scenario 4	100	88.9	0	8.0	8	8	8
Scenario 5	99.8	7.6	0.2	9.7	9	10	12
Scenario 6	99.5	4.8	0.5	10.8	10	11	14
Scenario 7	*85.2*	*46*	*14.8*	*16.2*	*14*	*16*	*17*
Scenario 8	*82.7*	*24.1*	*17*	*15.0*	*8*	*14*	*17*
Scenario 9	100	1.7	0	12.6	13	14	14
Scenario 10	100	0.9	0	15.2	16	17	17
Scenario 11	86.6	8.2	0.1	16.8	16	17	18
Scenario 12	99	33.5	1	11.2	11	12	13
Scenario 13	*94.9*	*26.1*	*5.1*	*8.1*	*8*	*8*	*10*
Scenario 14	*64.1*	*16.2*	*6.7*	*15.0*	*8*	*14*	*17*
Scenario 15	98.5	6.1	1.5	12.5	12	13	15
Scenario 16	99.8	3.9	0.2	12.0	11	12	14

Table C: Results from using Bootstrap duration CI method, but using standard fractional polynomials (mfp package in R) , with base-case design parameters (estimand=shortest duration non-inferior to 20 days within 10% risk difference). Acceptable and optimal power, Type-1 Error, Real minimum duration, minimum, 2.5% percentile and median duration recommended. In italics: scenarios for which type 1 error was not strictly controlled within 2.5%.

	Acceptable Power (%)	Optimal Power (%)	Type 1 Error (%)	True Min Duration	Estimated Minimum Duration	Estimated 2.5th Perc Duration	Estimated Median Duration
Scenario 1	98.7	32.4	1.3	13.1	12	14	15
Scenario 2	*95.6*	*44*	*4.4*	*14.5*	*14*	*14*	*16*
Scenario 3	*89.8*	*25.1*	*10.2*	*15.9*	*13*	*15*	*17*
Scenario 4	100	86.3	0	8.0	8	8	8
Scenario 5	100	0.4	0	9.7	10	11	12
Scenario 6	100	0.1	0	10.8	11	13	14
Scenario 7	*81.6*	*41.5*	*18.4*	*16.2*	*14*	*15*	*17*
Scenario 8	*57.6*	*13.1*	*42.4*	*15.0*	*8*	*12*	*16*
Scenario 9	100	1.6	0	12.6	13	14	15
Scenario 10	100	0.2	0	15.2	16	17	17
Scenario 11	100	0.2	0	16.8	17	18	18
Scenario 12	99.4	23.8	0.6	11.2	11	12	14
Scenario 13	*95.4*	*9.3*	*4.6*	*8.1*	*8*	*8*	*11*
Scenario 14	*60.4*	*16.6*	*39.6*	*15.0*	*8*	*12*	*16*
Scenario 15	99.8	4.3	0.2	12.5	12	13	14
Scenario 16	100	0.5	0	12.0	12	13	15

Table D: Results from using Bootstrap duration CI method, targeting a fixed cure rate estimand. Acceptable and optimal power, Type-1 Error, Real minimum duration, minimum, 2.5% percentile and median duration recommended. In italics: scenarios for which type 1 error was not strictly controlled within 2.5%.

	Acceptable Power (%)	Optimal Power (%)	Type 1 Error (%)	True Min Duration	Estimated Minimum Duration	Estimated 2.5th Perc Duration	Estimated Median Duration
Scenario 1	*96.3*	*13.7*	*3.2*	*13.1*	*12*	*13*	*16*
Scenario 2	99.4	13.1	0.5	14.5	14	15	16
Scenario 3	94.7	5.7	0.4	15.9	15	16	18
Scenario 4	100	87	0	8.0	8	8	8
Scenario 5	99.9	4.3	0.1	9.7	9	10	12
Scenario 6	99.9	2.4	0.1	10.8	10	12	13
Scenario 7	*93.9*	*36.3*	*3.3*	*16.2*	*15*	*16*	*18*
Scenario 8	*88.2*	*23.3*	*9.3*	*15.0*	*8*	*14*	*17*
Scenario 9	100	1.1	0	12.6	13	14	14
Scenario 10	100	6.1	0	15.2	16	16	17
Scenario 11	78.9	9.4	0.2	16.8	16	17	18
Scenario 12	99.9	48.3	0.1	11.2	11	12	13
Scenario 13	*97.2*	*43.8*	*2.8*	*8.1*	*8*	*8*	*10*
Scenario 14	*76.7*	*11.6*	*7.4*	*15.0*	*8*	*14*	*18*
Scenario 15	98.5	10.7	1.4	12.5	11	13	15
Scenario 16	100	5.8	0	12.0	12	12	13

Table E: Results from using Bootstrap duration CI method, targeting a fixed risk ratio estimand. Acceptable and optimal power, Type-1 Error, Real minimum duration, minimum, 2.5% percentile and median duration recommended. In italics: scenarios for which type 1 error was not strictly controlled within 2.5%.

	Acceptable Power (%)	Optimal Power (%)	Type 1 Error (%)	True Min Duration	Estimated Minimum Duration	Estimated 2.5th Perc Duration	Estimated Median Duration
Scenario 1	98	6.8	2	13.3	11	14	16
Scenario 2	99.8	13.5	0.2	14.7	14	15	16
Scenario 3	99.3	3.7	0.7	16.0	14	16	18
Scenario 4	100	84.1	0	8.0	8	8	8
Scenario 5	99.9	4.8	0.1	9.7	9	10	12
Scenario 6	*97*	*8.9*	*3*	*11.0*	*10*	*11*	*14*
Scenario 7	*96*	*42.2*	*4*	*16.4*	*15*	*16*	*18*
Scenario 8	*95.2*	*14.2*	*4.8*	*15.3*	*8*	*15*	*17*
Scenario 9	100	1.6	0	12.6	13	14	14
Scenario 10	100	0.5	0	15.2	16	17	17
Scenario 11	*95.5*	*64*	*4.5*	*17.1*	*17*	*17*	*18*
Scenario 12	99.6	32	0.4	11.3	11	12	13
Scenario 13	97.7	22.9	2.3	8.2	8	9	11
Scenario 14	*97.4*	*3.2*	*2.6*	*16.0*	*9*	*15*	*18*
Scenario 15	99.6	4.2	0.4	12.6	12	13	15
Scenario 16	*96.4*	*19.8*	*3.6*	*12.1*	*11*	*12*	*14*

Table F: Results from using Bootstrap duration CI method, targeting an acceptability frontier estimand. Acceptable and optimal power, Type-1 Error, Real minimum duration, minimum, 2.5% percentile and median duration recommended. In italics: scenarios for which type 1 error was not strictly controlled within 2.5%.

	Acceptable Power (%)	Optimal Power (%)	Type 1 Error (%)	True Min Duration	Estimated Minimum Duration	Estimated 2.5th Perc Duration	Estimated Median Duration
Scenario 1	98.7	3	1.3	14.8	12	15	18
Scenario 2	99.8	6.3	0.2	16.0	15	16	18
Scenario 3	96.3	14.9	1.8	17.6	16	18	19
Scenario 4	100	86.1	0	8.0	8	8	8
Scenario 5	99.9	2.9	0.1	9.9	9	10	13
Scenario 6	98	3.6	2	11.6	10	12	15
Scenario 7	99.3	27.9	0.7	17.8	17	18	19
Scenario 8	*92.2*	*28.4*	*7.5*	*17.3*	*8*	*17*	*19*
Scenario 9	100	0.2	0	12.7	13	14	15
Scenario 10	100	0	0	15.4	17	17	18
Scenario 11	86.9	6.2	0.3	17.8	17	18	19
Scenario 12	99.7	20.8	0.3	11.4	11	12	13
Scenario 13	*96.3*	*24.9*	*3.7*	*8.1*	*8*	*8*	*11*
Scenario 14	*47.1*	*5.2*	*4.4*	*17.8*	*8*	*17*	*19*
Scenario 15	99.3	2.7	0.7	13.6	12	14	17
Scenario 16	98.4	9.6	1.6	12.5	11	13	15

Table G: Results from using Bootstrap duration CI method, targeting a maximum gradient estimand. Acceptable and optimal power, Type-1 Error, Real minimum duration, minimum, 2.5% percentile and median duration recommended. In italics: scenarios for which type 1 error was not strictly controlled within 2.5%. Power is not available for scenario 14, for which the gradient is too steep even at the longest duration.

	Acceptable Power (%)	Optimal Power (%)	Type 1 Error (%)	True Min Duration	Estimated Minimum Duration	Estimated 2.5th Perc Duration	Estimated Median Duration
Scenario 1	*67*	*15.3*	*29.8*	*13.939*	*8*	*20*	*15*
Scenario 2	*62.5*	*30.3*	*36.9*	*16.848*	*12*	*20*	*17*
Scenario 3	*0*	*42.7*	*57.3*	*19.152*	*8*	*20*	*19*
Scenario 4	93.2	70.6	0	8	8	20	8
Scenario 5	*77.3*	*55*	*19*	*11.152*	*10*	*20*	*12*
Scenario 6	*90.1*	*32.3*	*4.7*	*11.636*	*8*	*20*	*13*
Scenario 7	*0*	*42.6*	*57.4*	*19.273*	*8*	*20*	*19*
Scenario 8	*12*	*42.8*	*57.2*	*18.788*	*8*	*20*	*17*
Scenario 9	23	0.1	0	13.818	14	20	20
Scenario 10	86.6	0	0	16.242	18	20	19
Scenario 11	*0*	*63.1*	*36.9*	*19.152*	*18*	*20*	*20*
Scenario 12	85	25.5	0.1	12.848	12	20	14
Scenario 13	*73.3*	*32*	*14*	*9.333*	*8*	*20*	*11*
Scenario 14	*NA*	*NA*	*52.7*	*20*	*8*	*20*	*18.5*
Scenario 15	*46*	*23.9*	*50.3*	*14.303*	*8*	*20*	*14*
Scenario 16	*46*	*36.7*	*27.1*	*14.061*	*13*	*20*	*15*

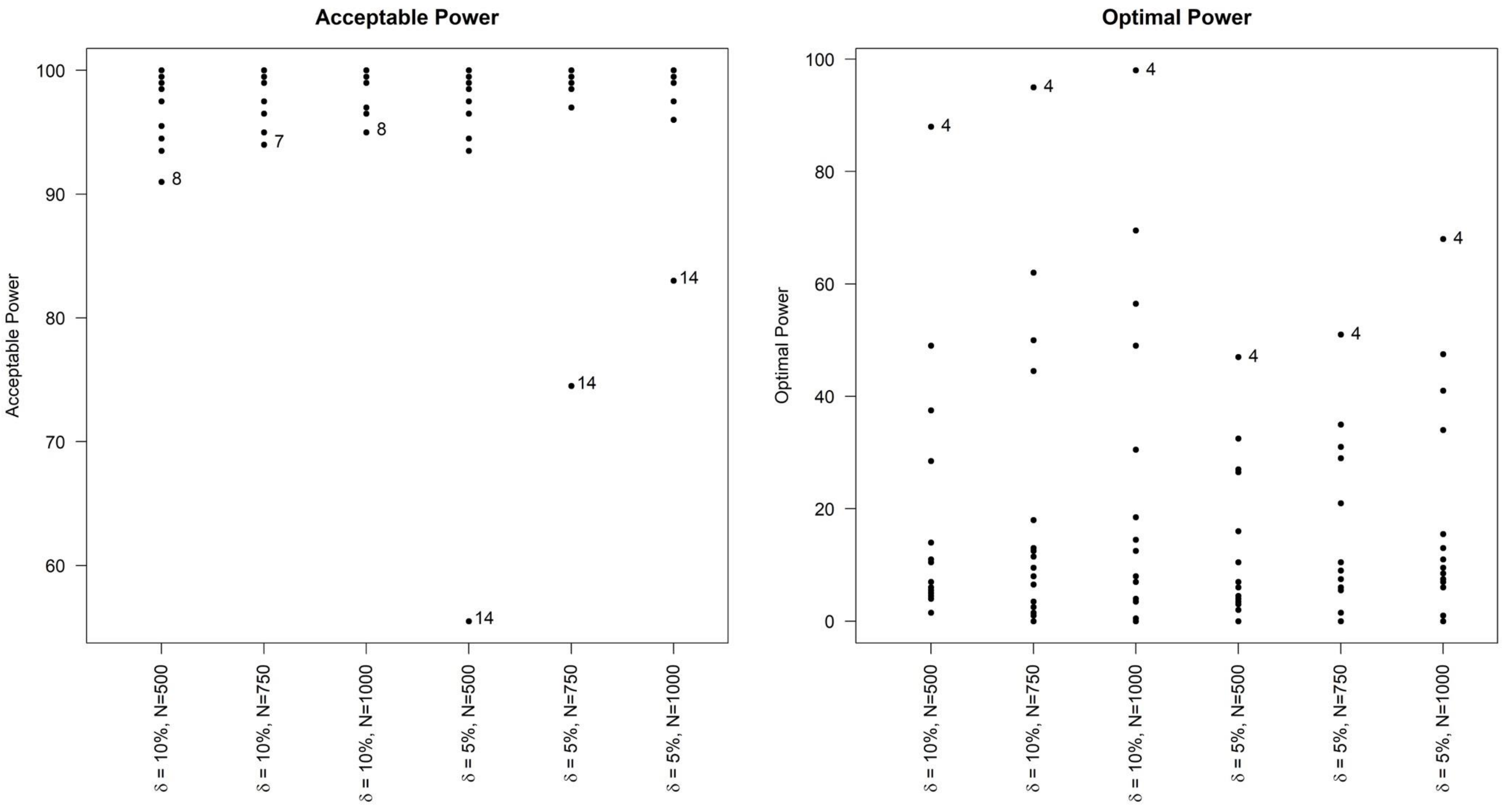

Figure A: Acceptable and Optimal Power using bootstrap duration CI with 6 different designs. The worst scenario in terms of acceptable power for each design and the best in terms of optimal power are indexed

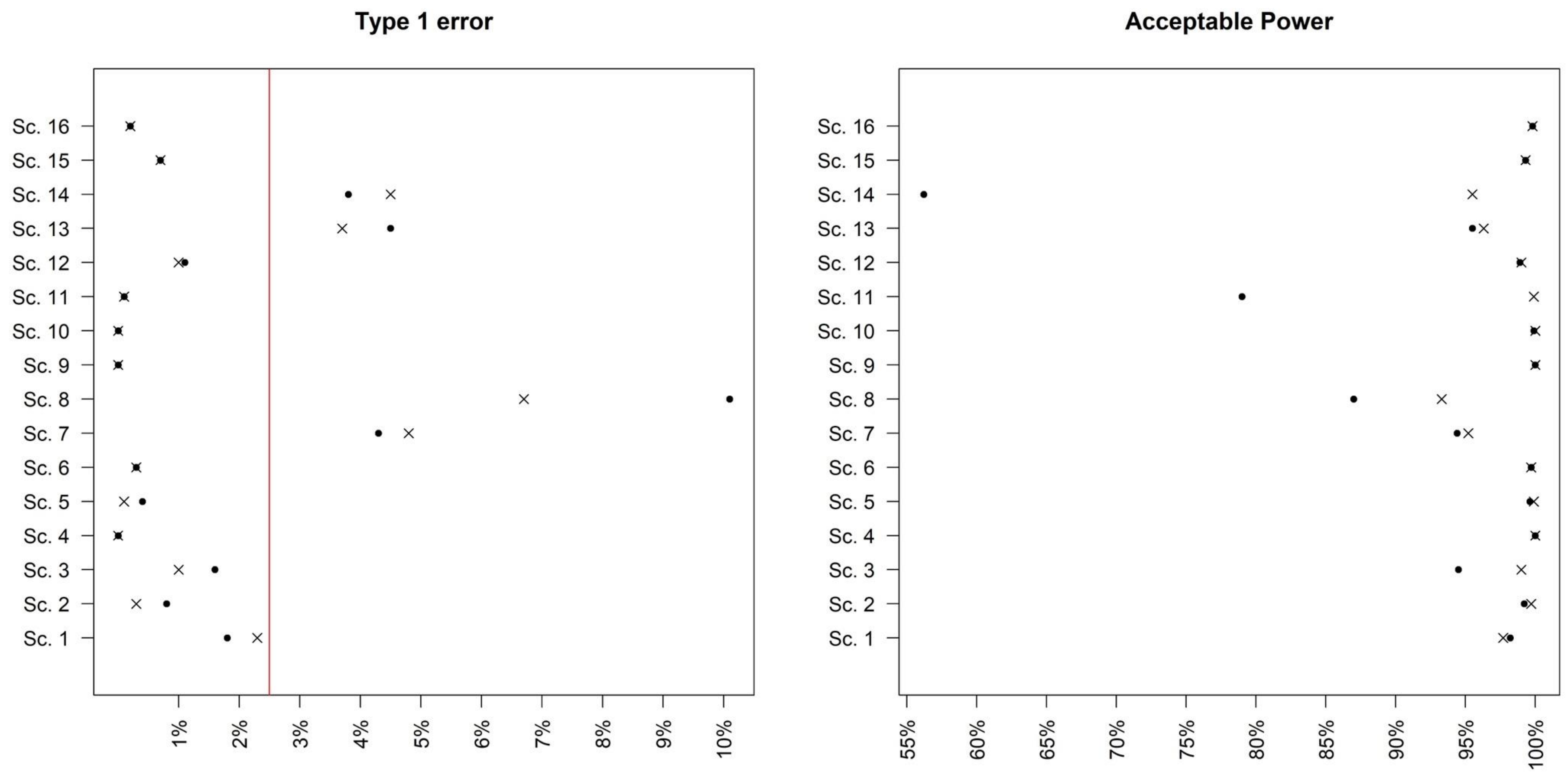

Figure b: Comparison of type 1 error rates (left panel) and acceptable power (right panel) across scenarios using Bootstrap CI method (·) and Bootstrap duration CI (X). Scenario 9-16 are those for which fractional polynomials do not include the correct model, and so we expect some degree of model mis-specification.

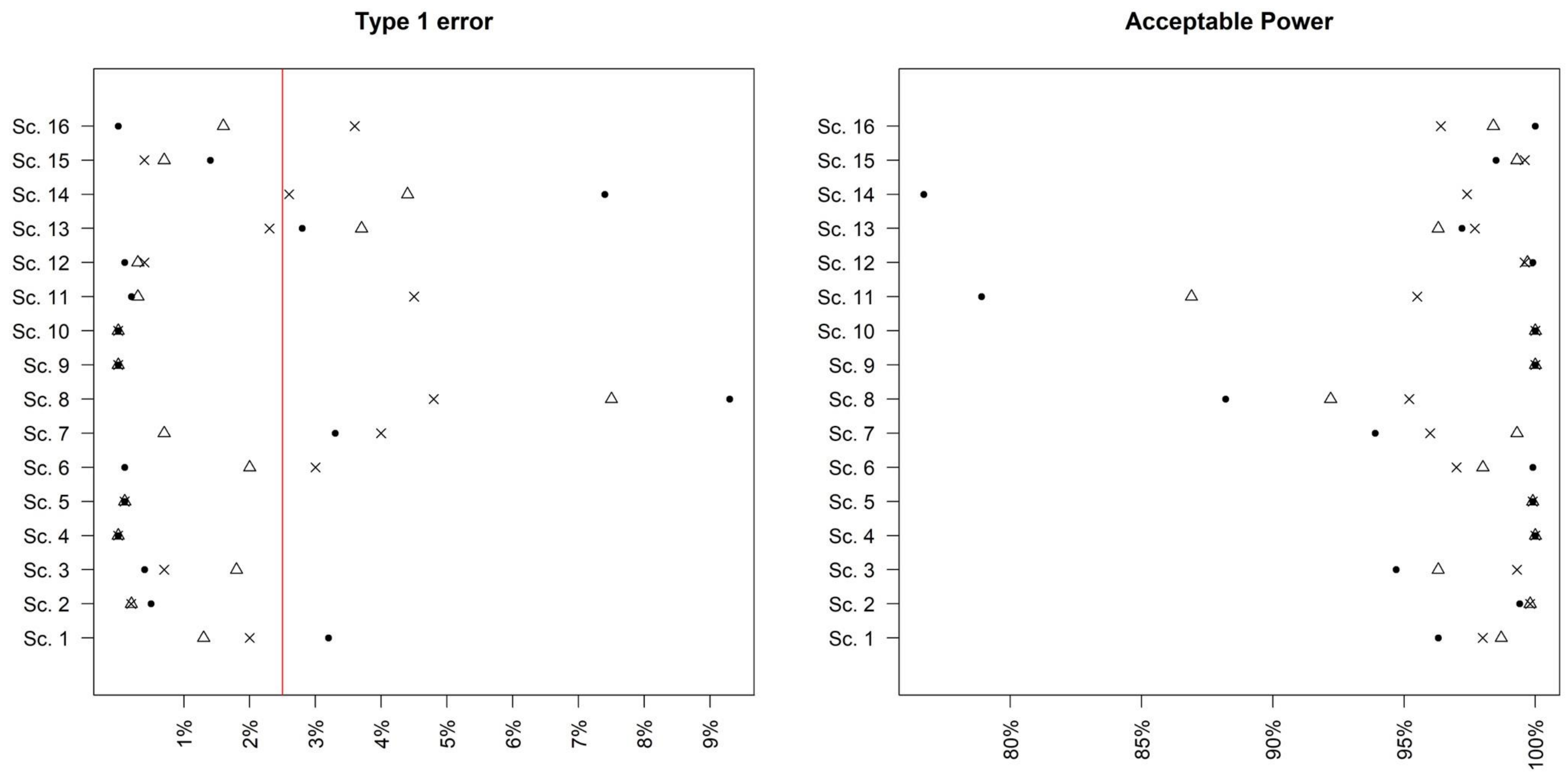

Figure c: Comparison of results of simulations using Bootstrap duration CI method across scenarios, with different estimation targets. Left panel shows type 1 error rates, right panel acceptable power. Dots represent fixed target rate, (X) fixed rate ratio and triangles the acceptability frontier target.

Table H: Simulation Scenarios.

Type / Equation	Plot	Type / Equation	Plot
1) Linear Duration-Response curve on log-odds scale: $$\pi = \frac{e^{0.85+0.17(D-8)}}{1+e^{0.85+0.17(D-8)}}$$	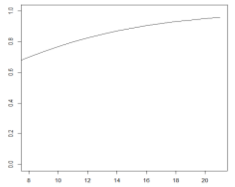	9) Logistic Growth Model – Early Growth: $$\pi = 0.05 + \frac{0.9}{1+e^{23-2D}}$$	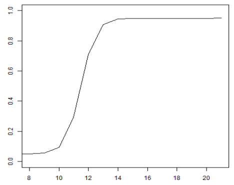
2) Quadratic + linear relation on log-odds scale: $$\pi = \frac{e^{0.62+0.13(D-8)+0.01(D-8)^2}}{1+e^{0.62+0.13(D-8)+0.01(D-8)^2}}$$	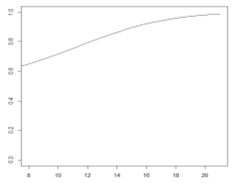	10) Logistic Growth Model – Later Growth: $$\pi = 0.05 + \frac{0.9}{1+e^{28-2D}}$$	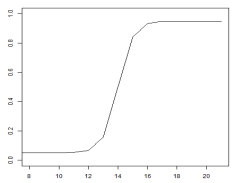
3) Quadratic relation on log-odds scale: $$\pi = \frac{e^{0.85+0.01(D-8)^2}}{1+e^{0.85+0.01(D-8)^2}}$$	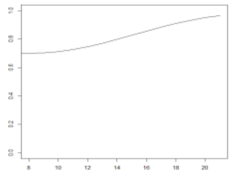	11) Gompertz Curve A: $$\pi = 0.9e^{-e^{-0.5(D-13)}}$$	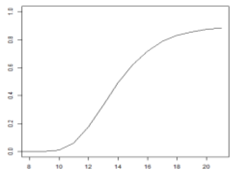
4) Constant response: $$\pi = 0.95$$	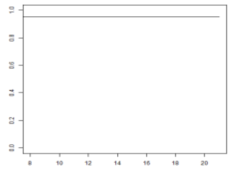	12) Gompertz Curve B: $$\pi = 0.9e^{-e^{-(D-9)}}$$	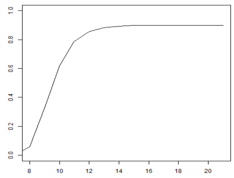
5) Logarithmic relation on log-odds scale: $$\pi = \frac{e^{0.85+1.19log(D-8)}}{1+e^{0.85+1.19log(D-8)}}$$	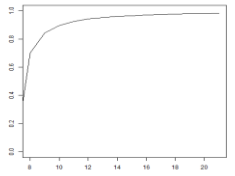	13) Gompertz Curve C: $$\pi = 0.9e^{-e^{-2(D-7)}}$$	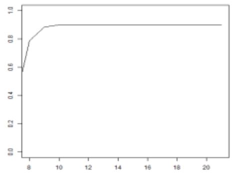
6) Square rooted relation on log-odds scale: $$\pi = \frac{e^{0.62+0.67\sqrt{D-8}}}{1+e^{0.62+0.67\sqrt{D-8})}}$$	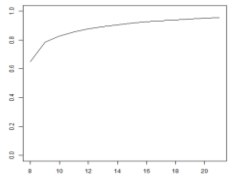	14) Quadratic on probability scale: $\pi = 0.7 + 0.01(D-8)^2$	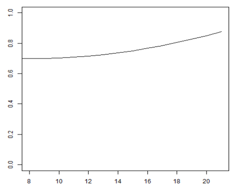
7) Cubic relation on log-odds scale: $$\pi = \frac{e^{1.10+0.002(D-8)^3}}{1+e^{1.10+0.002(D-8)^3}}$$	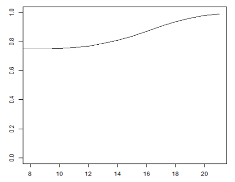	15) Quadratic on probability scale: $\pi = 0.7 - 0.01(D-8)^2 + 0.04(D-8)$	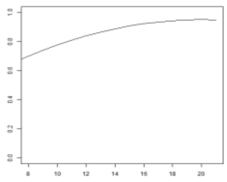
8) Cubic + Quadratic relation on log-odds scale: $$\pi = \frac{e^{1.39+0.002(D-8)^2+0.001(D-8)^3}}{1+e^{1.39+0.002(D-8)^2+0.001(D-8)^3}}$$	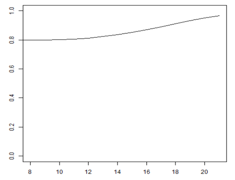	16) Linear Spline: D<11: $\pi = 0.5 + 0.10(D-8)$ D>11&D<14: $\pi = 0.8 + 0.04(D-11)$ D>14: $\pi = 0.94 + 0.01(D-14)$	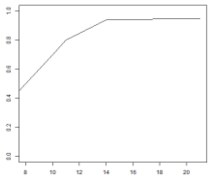

Appendix A. Delta Method Confidence Interval.

Suppose we ran our fractional polynomial algorithm estimating our duration-response curve:

$$\pi(D) = logit^{-1}(\hat{\alpha} + \hat{\beta}D + \hat{\gamma}D^2),$$

where a linear and a quadratic term have been selected by the algorithm, and $(\hat{\alpha}, \hat{\beta}, \hat{\gamma})$ is the vector of estimated model parameters. Our goal is to obtain a confidence interval for the difference in efficacy between two specific durations D_1 and D_2 . This can be written as a function of model parameters, as:

$$f(\alpha,\beta,\gamma) = \pi(D_1) - \pi(D_2) = logit^{-1}(\alpha + \beta D_1 + \gamma D_1^2) - logit^{-1}(\alpha + \beta D_2 + \gamma D_2^2).$$

Let $\mathbf{\Omega}$ be the variance covariance matrix of the three regression parameters, so that approximately:

$$\begin{pmatrix}\alpha\\ \beta\\ \gamma\end{pmatrix} \sim N\left(\begin{pmatrix}\hat{\alpha}\\ \hat{\beta}\\ \hat{\gamma}\end{pmatrix}, \mathbf{\Omega}\right)$$

And let the Jacobian of function $f(\alpha,\beta,\gamma)$ be the vector of partial derivatives with respect to the three variables:

$$\boldsymbol{J_f}(\alpha,\beta,\gamma) = \left(\frac{\partial}{\partial\alpha}f, \frac{\partial}{\partial\beta}f, \frac{\partial}{\partial\gamma}f\right) = \left(\frac{e^{\alpha+\beta D_1+\gamma D_1^2}}{(e^{\alpha+\beta D_1+\gamma D_1^2})^2} - \frac{e^{\alpha+\beta D_2+\gamma D_2^2}}{(e^{\alpha+\beta D_2+\gamma D_2^2})^2}\right.$$

$$\left., D_1\frac{e^{\alpha+\beta D_1+\gamma D_1^2}}{(e^{\alpha+\beta D_1+\gamma D_1^2})^2} - D_2\frac{e^{\alpha+\beta D_2+\gamma D_2^2}}{(e^{\alpha+\beta D_2+\gamma D_2^2})^2}, D_1^2\frac{e^{\alpha+\beta D_1+\gamma D_1^2}}{(e^{\alpha+\beta D_1+\gamma D_1^2})^2} - D_2^2\frac{e^{\alpha+\beta D_2+\gamma D_2^2}}{(e^{\alpha+\beta D_2+\gamma D_2^2})^2}\right)$$

Then, using the delta method, we have that:

$$f(\alpha,\beta,\gamma) = \pi(D_1) - \pi(D_2) \xrightarrow{d} N\left(\ f(\hat{\alpha},\hat{\beta},\hat{\gamma}),\ \ \boldsymbol{J_f}(\hat{\alpha},\hat{\beta},\hat{\gamma})\mathbf{\Omega}\boldsymbol{J_f}(\hat{\alpha},\hat{\beta},\hat{\gamma})^T\ \right)$$
